# Using CRISPR/Cas9 genome editing system to create *MaGA20ox2* gene‐modified semi‐dwarf banana

**DOI:** 10.1111/pbi.13216

**Published:** 2019-08-20

**Authors:** Xiuhong Shao, Shaoping Wu, Tongxin Dou, Haocheng Zhu, Chunhua Hu, Heqiang Huo, Weidi He, Guiming Deng, Ou Sheng, Fangcheng Bi, Huijun Gao, Tao Dong, Chunyu Li, Qiaosong Yang, Ganjun Yi

**Affiliations:** ^1^ Key Laboratory of South Subtropical Fruit Biology and Genetic Resource Utilization (Ministry of Agriculture and Rural Affairs) Guangdong Key Laboratory of Tropical and Subtropical Fruit Tree Research Institute of Fruit Tree Research Guangdong Academy of Agricultural Sciences Guangzhou China; ^2^ Horticulture and Landscape College Hunan Agricultural University Changsha China; ^3^ State Key Laboratory of Plant Cell and Chromosome Engineering Center for Genome Editing Institute of Genetics and Developmental Biology Chinese Academy of Sciences Beijing China; ^4^ University of Chinese Academy of Sciences Beijing China; ^5^ Mid‐Florida Research and Education Center University of Florida Apopka FL USA

**Keywords:** CRISPR/Cas9, semi‐dwarf, *MaGA20ox2*, banana

Banana is an important staple crop and widely grown in more than 130 countries around the world. Plant height is one of the major characteristics of crops (Wang *et al*., 2018); however, the height of commercial banana varieties is more than 2 m, and higher varieties are frequently challenged by the weak lodging and severe damages caused by typhoons and storms. Dwarf varieties are suitable for mechanized plant maintenance and fruit harvesting (Dash and Rai, 2016); thus, development of semi‐dwarf and dwarf banana is important to current farming systems.

Gibberellin (GA) is one of the most important determinants of plant height (Sasaki *et al*., 2002), and mutations in genes for biosynthesis or signal transduction of GAs generally result in dwarf phenotypes. For example, a 383 bp deletion in the *OsGA20ox2* caused a premature stop codon and lower bioactive GA production, resulting in the semi‐dwarf phenotype in IR8 *sd1* mutant (Sasaki *et al*., 2002; Spielmeyer *et al*., 2002). Similarly, Chen *et al*. (2016) found that the dwarf phenotype of banana mutant ‘8818‐1’ was mainly derived from differential expression in 6 genes regulating GA production compared with its wild‐type Williams 8818. In this study, we found five *GA20ox2* homologous genes in banana cultivar ‘Gros Michel’ (*Musa acuminate*, AAA group). Real‐time quantitative PCR results showed that these genes specifically expressed in certain tissues, such as *Ma11g17210* and *Ma06g27710* exhibited highest mRNA level in leaves, while *Ma11 g10500* was highly expressed in fruit peels. By contrast, *Ma04g15900* exhibited higher transcript level in fruit axis, peel and pulp, and *Ma08g32850* was much lower in spite of its similar expression pattern to *Ma04g15900* (Figure 1a). To date, the CRISPR/Cas9 system has been extensively used for generating targeted mutations in crop genomes for functional analysis and plant precision breeding (Feng *et al*., 2018; Hu *et al*., 2017). To examine the functions of five *MaGA20ox2* genes in ‘Gros Michel’, we selected two sgRNAs that specifically target the second exon of each gene (Figure 1b), and these two sgRNAs were separately driven by an U6a or U3 promoter. In addition, a *GFP* gene was fused with *HPT* of the pYLCRISPR/Cas9P_ubi_‐H vector (Ma *et al*., 2015) to form a pCRISPR/Cas9‐GA2T expression plasmid (Figure 1c) which was transformed into *Agrobacterium* strain EHA105.

**Figure 1:**
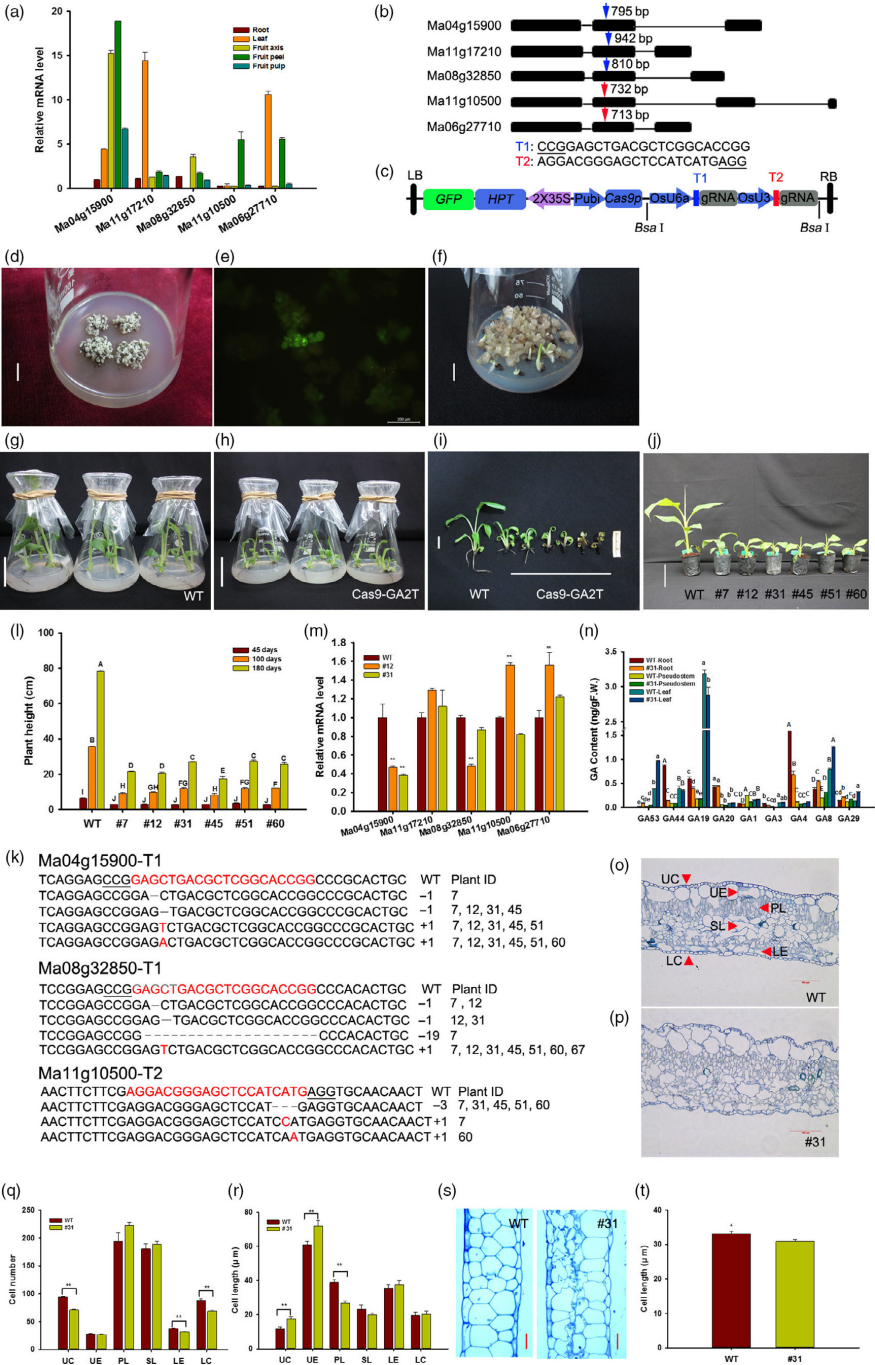
CRISPR/Cas9‐induced mutations in *MaGA20ox2* regulate semi‐dwarfism in banana. (a) Expression pattern of five *MaGA20ox2* genes in different tissues. (b) Two sgRNAs (T1 in blue arrow and T2 in red) were selected for targeting the second exon of *MaGA20ox2* genes. The PAM sequences were underlined. (c) Schematic illustration of the CRISPR/Cas9 construct with two sgRNA cassettes and a modified selection marker *HPT‐GFP*. (d) Cell clusters cultured on SSM to form resistant somatic embryos. Bar = 2 cm. (e) Cell clusters with stable green fluorescence of GFP. Bar = 200 μm. (f) Germinated budlets from somatic embryos. Bar = 2 cm. WT (g) and transgenic plantlets (h) on rooting medium without hygromycin B. Bar = 6 cm. Phenotype of WT and transgenic lines from rooting medium for 30 days (i) (Bar = 1.5 cm) and grown in pots for 60 days (j) (Bar = 10 cm). (k) Different types of CRISPR/Cas9‐induced mutations detected in *Ma04 g15900*,* Ma08 g32850* and *Ma11 g10500* genes of mutants. The numbers of insertions and deletions in T1 and T2 of mutants and plant IDs were shown in right. (l) Plant height of mutants and WT plants after 45, 100 and 180 days grown in greenhouse. (m) The expression of *MaGA20ox2* genes in pseudostems of mutants and WT plants. (n) Quantification of endogenous GAs in different tissues of WT and #31. Cross‐sectioning images of the middle leaves of WT (o) and #31 (p). Arrows indicate each type of cells, UC, upper corneum; UE, upper epidermis; PL, palisade layer; SL, spongy layer; LE, lower epidermis; and LC, lower corneum. Bar = 100 μm. Cell number (q) and cell length (r) of different types of cells in WT and #31 leaves. (s) Longitudinal sectioning images of thinner parts of petioles of WT and #31. Bar = 20 μm. (t) Cell length in petioles of WT and #31. The upper case (l, n, *P *<* *0.01, ANOVA), lower case (n, *P *<* *0.05, ANOVA) and asterisks (m, q and r, **P <* *0.01; t, *P <* *0.05, Student's *t*‐test) indicate the statistical significance between mutants and WT plants.

Genetic transformation of ‘Gros Michel’ was performed using embryogenic cell suspension. The transformed cell clusters were screened on semi‐solid selection medium (SSM) containing hygromycin B (Figure 1d), and ones with strong GFP fluorescence were selected for somatic embryo induction and germination on SSM (Figures 1e,f). One hundred and fifty‐two independent transgenic lines were obtained in this study, most of which exhibited sparse brownish roots on the rooting medium without hygromycin B (Figure 1h,i). Interestingly, the nontransformed control plants rooted normally (Figure 1g,i), indicating that GA may mediate root development. Healthy transgenic plants were transplanted and maintained in the greenhouse (Figure 1j).

To confirm the mutations induced by the CRISPR/Cas9 system, we amplified the target regions of transformed and wild‐type (WT) plants and performed high‐throughput sequencing. The results showed that 7 mutant lines with semi‐dwarf phenotype contained different types of mutations (Figure 1k). In the T1 site of *Ma04g15900* and *Ma08g32850*, the short insertions (+1) were the much common mutation in seven mutants, and short deletions (−1) appeared in #7, #12, #31 and #45, while the 19‐bp deletion was only found in #7. In the T2 site of *Ma11g10500*, short deletion (−3) appeared in #7, #31, #45, #51 and #60, and short insertions (+1) were only found in #7 or #60 (Figure 1k). Furthermore, we found that mutations in *Ma04g15900* and/or *Ma08g32850* genes may play important roles for semi‐dwarf phenotype in ‘Gros Michel’. These results indicated that the CRISPR/Cas9‐mediated system is efficient for precise genome editing in banana.

To investigate the changes in plant height, we measured the height of six mutant lines and found that it was significantly decreased compared with the WT at three developmental stages (Figure 1l). To understand how these mutations affect the *MaGA20ox2* genes, the transcript level of these five genes in banana pseudostems was performed. Compared with WT, the transcript level of *Ma04g15900* and *Ma08g32850* was significantly down‐regulated, while *Ma11g10500* and *Ma06g27710* was significantly up‐regulated in #12, and that *Ma04g15900* was significantly down‐regulated in #31 (Figure 1m). To confirm whether GA biosynthesis was impaired in mutants, endogenous GAs of different tissues were quantified. The results showed a higher content of GA_53_ and lower content of GA_44_, GA_19_, GA_20_ in #31 compared with WT. Also, the amounts of active GA_1_, GA_3_ and GA_4_ were decreased in roots and pseudostems of #31, while the content of inactive GA_8_ and GA_29_ was much higher (Figure 1n).

To our surprise, mutant lines had thicker and dark greener leaves than WT and that was similar to *Arabidopsis* (Peng and Harberd, 1997). We analysed the differences in middle leaves and petioles of #31 and WT at 100 days after transplanting. The results showed that corneum and epidermal cells of WT were regular and smooth (Figure 1o), while these cells of #31 were irregular (Figure 1p). The cell numbers of upper corneum (UC), lower epidermis and lower corneum of #31 were significantly reduced compared with WT (Figure 1q). In addition, the alterations in cell length were different from changes in cell number. The cell length of UC and upper epidermis was longer and that palisade layer was shorter in #31 than that of WT (Figure 1r). These results may explain why the leaves of mutants were thicker than the controls. Furthermore, longitudinal sections of the thinner parts of petioles showed that cells of WT were regular, while inner cells of #31 were partially irregular (Figure 1s). In addition, the cell length of #31 was shorter than WT (Figure 1t). These results of plant height and leaf cell structures were consistent with *Carrizo citrange* (Fagoaga *et al*., 2007) and provide a new insight into understanding the roles of *MaGA20ox2* genes in plant architecture.

In summary, we have successfully applied the CRISPR/Cas9 system to edit the *MaGA20ox2* genes in ‘Gros Michel’ and obtained semi‐dwarf mutants. Manipulation of *Ma04g15900* and/or *Ma08g32850* genes is likely to be an efficient strategy to develop semi‐dwarf or dwarf banana germplasm resources. Future efforts will be given to evaluate the characteristics of growth and yield of these mutants at multiple locations in different years to minimize the environmental influences. Results from these current and future studies will be of significant impact on banana dwarf breeding.

## Conflict of interest

The authors declare no conflict of interest.
